# Uniform Convergence of Cesaro Averages for Uniquely Ergodic C^*^-Dynamical Systems

**DOI:** 10.3390/e20120987

**Published:** 2018-12-19

**Authors:** Francesco Fidaleo

**Affiliations:** Dipartimento di Matematica, Università di Roma “Tor Vergata”, Via della Ricerca Scientifica 1, 00133 Roma, Italy; fidaleo@mat.uniroma2.it

**Keywords:** ergodic theorems, C*-dynamical systems

## Abstract

Consider a uniquely ergodic $C^{*}$-dynamical system based on a unital *-endomorphism Φ of a $C^{*}$-algebra. We prove the uniform convergence of Cesaro averages $\frac{1}{n}\sum_{k=0}^{n-1}\lambda^{-n}\Phi \left(a\right)$ for all values λ in the unit circle, which are not eigenvalues corresponding to “measurable non-continuous” eigenfunctions. This result generalizes an analogous one, known in commutative ergodic theory, which turns out to be a combination of the Wiener–Wintner theorem and the uniformly convergent ergodic theorem of Krylov and Bogolioubov.

## 1. Introduction

Motivated by the question of justifying the thermodynamical laws with the microscopic principles of statistical mechanics (i.e., the so-called ergodic hypothesis), the investigation of the ergodic properties of classical (i.e., commutative) dynamical systems has a long history.

Indeed, given a classical dynamical system (X,T,μ), where *X* is a compact space, T:X→X a continuous map, and finally, μ an invariant probability measure under the natural action of *T*, the classical ergodic theory primarily deals with the long time behavior of the Cesaro means (ergodic averages)

$$M_{n}\left(f\right):=\frac{1}{n}\sum_{k=0}^{n-1}f\circ T^{k}\;,\;f\in C\left(X\right)\;,\;\;n\in N\;,$$

of continuous functions, or more generally of any measurable function *f* w.r.t. the σ-algebra generated by the μ-measurable sets.

Among the most famous classical ergodic theorems, we mention the Birkhoff individual ergodic theorem concerning the study of the point-wise limit $\lim_{n\to +\infty }M_{n}\left(f\right)\left(x\right)$ and the von Neumann mean ergodic theorem concerning the limit $L^{2}\;-\;\lim_{n\to +\infty }M_{n}\left(f\right)$, whenever *f* is square-summable.

The quantity of results obtained in the commutative setting is too huge to summarize an exhaustive description. However, a standard reference, dealing mainly with the classical case, is [1]. We also mention several unconventional ergodic theorems (e.g., [2]), which play a fundamental role in number theory.

Most of the known ergodic results concern the so-called $W^{*}$-setting, which roughly speaking involve the functions in the commutative $W^{*}$-algebra $L^{\infty }\left(X,d\;\mu \right)=\pi_{\mu }{\left(C\left(X\right)\right)}^{\prime \prime }$; see, e.g., Theorem III.1.2 in [3].

At the same way, also the investigation of the uniform convergence of ergodic averages (i.e., involving directly continuous functions in the commutative $C^{*}$-algebra C(X)) is of great interest.

Among such kind of results, we mention the following one relative to the so-called uniquely ergodic dynamical systems. The classical dynamical system (X,T) is said to be uniquely ergodic if there exists a unique probability Radon measure μ, which is invariant under the action of the transformation *T*. It was proven in [4] that (X,T) is uniquely ergodic if and only if, for the Cesaro average of any f∈C(X),

$$\lim_{n}\frac{1}{n}\sum_{k=0}^{n-1}f\circ T^{k}=\int_{X}fd\;\mu \;,$$

uniformly. In [5], the last result was generalized to averages of the form

$$\lim_{n}\frac{1}{n}\sum_{k=0}^{n-1}\lambda^{-k}f\circ T^{k}\;,$$

for certain λ in the unit circle T.

With the impetuous growth of quantum physics, it was natural to address the systematic investigation of ergodic properties of quantum (i.e., noncommutative) dynamical systems. On the other hand, the situation in the quantum setting appears rather more complicated than the classical situation. Typically, one must provide all statements in terms of the dual concept of “functions” instead of “points”. Therefore, algebras of functions are replaced by general $C^{*}$ or $W^{*}$-algebras A, and the action on functions $\Phi_{T}\left(f\right):=f\circ T$ of the transformation *T* is replaced by that of a positive linear map Φ:A→A acting directly on elements of A. Concerning some general ergodic properties of noncommutative dynamical systems, the reader is referred to [6] and the literature cited therein.

The systematic study of some natural generalizations of ergodic properties to the quantum case has been carried out in the seminal paper [7]. The reader is also referred to [8,9,10,11] for some quantum versions of unconventional (called also “entangled”) ergodic theorems and to [12,13,14] for the investigation of the strong ergodic properties of dynamical systems arising from free probability and generalizing the unique ergodicity. Some natural applications of ergodic results to quantum probability are also carried out; see [15] and the references cited therein.

The goal of the present note is to provide the quantum generalization of the interesting result proven in [5] involving the uniform convergence of Cesaro averages relative to uniquely ergodic quantum dynamical systems “continuous” eigenfunctions. This result can be considered a combination of the Wiener–Wintner theorem (cf. [16]) and the uniformly convergent ergodic theorem of Krylov and Bogolioubov (cf. [4]).

More precisely, let (A,Φ,φ) be a uniquely ergodic $C^{*}$-dynamical system based on a unital $C^{*}$-algebra and a unital *-homomorphism Φ:A→A with φ∈S(A) as the unique invariant state. Consider the covariant Gelfand–Naimark–Segal representation $\left(H_{\varphi },\pi_{\varphi },V_{\varphi ,\Phi },\xi_{\varphi }\right)$ associated with the state φ, together with the peripheral pure-point spectra (see below for the definition) $\sigma_{\mathrm{pp}}^{\mathrm{ph}}\left(\Phi \right)$ and $\sigma_{\mathrm{pp}}^{\mathrm{ph}}\left(V_{\varphi ,\Phi }\right)$ of Φ and the isometry $V_{\varphi ,\Phi }\in B\left(H_{\varphi }\right)$, respectively. We see that $\sigma_{\mathrm{pp}}^{\mathrm{ph}}\left(\Phi \right)\subset \sigma_{\mathrm{pp}}^{\mathrm{ph}}\left(V_{\varphi ,\Phi }\right)$, but in general, they are different. Put for a∈A and λ∈T,

(1) $$M_{a,\lambda }\left(n\right):=\frac{1}{n}\sum_{k=0}^{n-1}\lambda^{-k}\Phi^{k}\left(a\right)\;,\;n\in N\;.$$

We show that, in the norm topology of A (compare with Proposition 3.2 in [7]),

(i) if $\lambda \in \sigma_{\mathrm{pp}}^{\mathrm{ph}}\left(\Phi \right)$, then $M_{a,\lambda }\left(n\right)\to \varphi \left(u_{\lambda }^{*}a\right)u_{\lambda }$, where $u_{\lambda }\in A$ is a unitary eigenvector (i.e., a “continuous eigenfunction” in the language of [5]) corresponding to λ, which is uniquely determined up to a phase-factor;
(ii) if $\lambda \in \sigma_{\mathrm{pp}}^{\mathrm{ph}}{\left(V_{\varphi ,\Phi }\right)}^{c}$ (i.e., λ does not admit any nontrivial “measurable eigenfunction” in the language of [5]), then $M_{a,\lambda }\left(n\right)\to 0$.

We end the paper with some example based on the tensor product, which is however nontrivial, of an Anzai skew product (cf. [17]) and a uniquely mixing noncommutative dynamical system, for which the sequence ${\left\{M_{a,\lambda }\left(n\right)\right\}}_{n\in N}$ does not converge for some a∈A and $\lambda \in \sigma_{\mathrm{pp}}^{\mathrm{ph}}\left(V_{\varphi ,\Phi }\right)\backslash \sigma_{\mathrm{pp}}^{\mathrm{ph}}\left(\Phi \right)$.

## 2. Preliminaries

With T:={λ∈C∣|λ|=1}, we denote the unit circle of the complex plane. It is homeomorphic to the interval [0,2π) by $\theta \in \left[0,2\pi \right)\mapsto e^{-ı\theta }$, after identifying the end-points zero and 2π.

A (discrete) $C^{*}$-dynamical system is a triplet (A,Φ,φ) consisting of a $C^{*}$-algebra, a positive map Φ:A→A, and a state φ∈S(A) such that φ∘Φ=φ. Consider the Gelfand–Naimark–Segal (GNS for short) representation $\left(H_{\varphi },\pi_{\varphi },\xi_{\varphi }\right)$; see, e.g., [3]. If in addition

$$\varphi \left(\Phi {\left(a\right)}^{*}\Phi \left(a\right)\right)\leq \varphi \left(a^{*}a\right)\;,\;a\in A\;,$$

then there exists a unique linear contraction $V_{\varphi ,\Phi }\in B\left(H_{\varphi }\right)$ such that $V_{\varphi ,\Phi }\xi_{\varphi }=\xi_{\varphi }$ and

$$V_{\varphi ,\Phi }\pi_{\varphi }\left(a\right)\xi_{\varphi }=\pi_{\varphi }\left(\Phi \left(a\right)\right)\xi_{\varphi }\;,\;a\in A\;.$$

The quadruple $\left(H_{\varphi },\pi_{\varphi },V_{\varphi ,\Phi },\xi_{\varphi }\right)$ is called the covariant GNS representation associated with the triplet (A,Φ,φ).

If Φ is multiplicative, hence a *-homomorphism, then $V_{\varphi ,\Phi }$ is an isometry with final range $V_{\varphi ,\Phi }V_{\varphi ,\Phi }^{*}$, the orthogonal projection onto the subspace $\bar{\pi_{\varphi }\left(\Phi \left(A\right)\right)\xi_{\varphi }}$; see, e.g., Lemma 2.1 of [7].

For the $C^{*}$-dynamical system (A,Φ,φ), the case when A is a unital $C^{*}$-algebra with unity $1\;\;I\equiv 1\;\;I_{A}$, and Φ is multiplicative and identity-preserving, i.e., a unital *-homomorphism, is of primary importance. Indeed, denote by $A^{\Phi }:=\left\{a\in A|\Phi (a)=a\right\}$ the fixed-point subalgebra, and

$$\sigma_{\mathrm{pp}}^{\mathrm{ph}}\left(\Phi \right):=\left\{\lambda \in T|\lambda \;\mathrm{is}\;\mathrm{an}\;\mathrm{eigenvalue}\;\mathrm{of}\;\Phi \right\}$$

the set of the peripheral eigenvalues of Φ (i.e., the peripheral pure-point spectrum), with $A_{\lambda }$ the relative eigenspaces. Obviously, $1\;\;I\in A^{\Phi }=A_{1}$.

For $\xi \in H_{\varphi }$ and n∈Z, consider the sequence

$$\hat{\mu_{\xi }}\left(n\right):=\{\begin{array}{cc} \langle V_{\varphi ,\Phi }^{n}\xi ,\xi \rangle & \mathrm{if}\;\;n\geq 0\;,\\ \bar{\langle V_{\varphi ,\Phi }^{n}\xi ,\xi \rangle } & \mathrm{if}\;\;n<0\;. \end{array}$$

**Proposition** **1.** *For each $\xi \in H_{\varphi }$, the sequence ${\left\{\hat{\mu_{\xi }}\left(n\right)\right\}}_{n\in Z}$ is positive definite, and therefore, it is the Fourier transform of a positive bounded Radon measure $\mu_{\xi }$ on the unit circle T.*

**Proof.**  Since Φ is multiplicative, $V_{\varphi ,\Phi }$ is an isometry. Consider its Sz-Nagy dilation (cf. [18])

$$V=\left(\begin{array}{cc} V_{\varphi ,\Phi } & I_{H_{\varphi }}-V_{\varphi ,\Phi }V_{\varphi ,\Phi }^{*}\\ 0 & -V_{\varphi ,\Phi }^{*} \end{array}\right)$$

acting on the direct sum $H_{\varphi }⊕H_{\varphi }$, together with its spectral resolution (e.g., [19])

$$V=\int_{0}^{2\pi }e^{-ın\theta }d\;E\left(\theta \right)\;,$$

and finally the vector

$$\eta_{\xi }=\left(\begin{array}{c} \xi \\ 0 \end{array}\right)\in H_{\varphi }⊕H_{\varphi }\;.$$

Notice that

$$\hat{\mu_{\xi }}\left(n\right)=\int_{0}^{2\pi }e^{-ın\theta }d\;\;\langle E\left(\theta \right)\eta_{\xi },\eta_{\xi }\rangle \;,$$

and therefore, $\hat{\mu_{\xi }}\left(n\right)$ is the Fourier transform of the positive bounded Radon measure $\mu_{\xi }=d\;\;\langle E\left(\theta \right)\eta_{\xi },\eta_{\xi }\rangle$ on the unit circle. □

Consider the pure-point peripheral spectrum

$$\sigma_{\mathrm{pp}}^{\mathrm{ph}}\left(V_{\varphi ,\Phi }\right):=\left\{\lambda \in T|\lambda \;\mathrm{is}\;\mathrm{an}\;\mathrm{eigenvalue}\;\mathrm{of}\;V_{\varphi ,\Phi }\right\}$$

of $V_{\varphi ,\Phi }$. Denote with $P_{\lambda }\in B\left(H_{\varphi }\right)$ the orthogonal projection onto the eigenspace generated by the eigenvectors associated with λ∈T, with the convention $P_{\lambda }=0$ if $\lambda \notin \sigma_{\mathrm{pp}}^{\mathrm{ph}}\left(V_{\varphi ,\Phi }\right)$.

With an abuse of language, $\mu_{\xi }$ is the spectral measure of $V_{\varphi ,\Phi }$ relative to $\xi \in H_{\varphi }$. Therefore, if $\lambda =e^{-ı\theta }\in T$, then $\mu_{\xi }\left(\left\{\theta \right\}\right)={∥P_{\lambda }\xi ∥}^{2}$.

The $C^{*}$-dynamical system (A,Φ) made of a unital $C^{*}$-algebra A and an identity-preserving completely positive map Φ:A→A is said to be uniquely ergodic if there exists only one invariant state φ for the dynamics induced by Φ. For a uniquely ergodic $C^{*}$-dynamical system, we simply write (A,Φ,φ) by pointing out that φ∈S(A) is the unique invariant state.

From now on, we specialize the situation to the case when Φ is a unital *-homomorphism of the unital $C^{*}$-algebra A.

For the sake of completeness, we collect some standard results, which are probably known to the experts.

**Proposition** **2.** Let the $C^{*}$-dynamical system (A,Φ,φ) be uniquely ergodic. Then, $\sigma_{\mathrm{pp}}^{\mathrm{ph}}\left(\Phi \right)$ is a subgroup of T, and all corresponding eigenspaces $A_{\lambda }$, $\lambda \in \sigma_{\mathrm{pp}}^{\mathrm{ph}}\left(\Phi \right)$, have dimension one and are generated by a single unitary $u_{\lambda }$.

**Proof.**  Since (A,Φ,φ) is uniquely ergodic, we have for the ergodic average,

$$\frac{1}{n}\sum_{k=0}^{n-1}\Phi^{k}\left(a\right)⟶\varphi \left(a\right)1\;\;I\;,\;a\in A\;,$$

uniformly, where φ is the unique invariant state. Suppose $a\in A^{\Phi }$. We get $a=\frac{1}{n}\sum_{k=0}^{n-1}\Phi^{k}\left(a\right)⟶\varphi \left(a\right)1\;\;I$, and thus, *a* is a multiple of the identity.

Fix now $\lambda \in \sigma_{\mathrm{pp}}^{\mathrm{ph}}\left(\Phi \right)$ and $a,b\in A_{\lambda }\backslash \left\{0\right\}$. Then, $a^{*}b=\alpha 1\;\;I$ and $ba^{*}=\beta 1\;\;I$ for some numbers α,β. Suppose α=0. Since $aa^{*}$ is a non-null multiple, say *c*, of the identity, we have $aa^{*}b=0$, which means b=0, a contradiction. At the same way, we verify $ba^{*}\neq 0$. Now, $\alpha^{-1}a^{*}$ and $\beta^{-1}a^{*}$ are left and right inverses of *b*. This means that *b* is invertible and $b^{-1}=\alpha^{-1}a^{*}$. At the same way, *a* is invertible, as well. Moreover, $ab^{-1}=\alpha^{-1}aa^{*}=\alpha^{-1}cI$. This means $a=\alpha^{-1}cb$, that is *a* is a multiple of *b*. Since $aa^{*}=cI$, we argue that $A_{\lambda }=Cu_{\lambda }$ for the unitary $u_{\lambda }=c^{-1/2}a$.

Let now $\lambda_{j}\in \sigma_{\mathrm{pp}}^{\mathrm{ph}}\left(\Phi \right)$ with $u_{\lambda_{j}}$ unitaries in $A_{\lambda_{j}}$, j=1,2. First, $u_{\lambda_{j}}^{*}$ is a unitary eigenvector corresponding to $\lambda_{j}^{-1}$ because Φ is a real map. Second, $u_{\lambda_{1}}u_{\lambda_{2}}$ is a unitary eigenvector corresponding to $\lambda_{1}\lambda_{2}$ because Φ is multiplicative. □

**Corollary** **1.** Let the $C^{*}$-dynamical system (A,Φ,φ) be uniquely ergodic. Then, $\sigma_{\mathrm{pp}}^{\mathrm{ph}}\left(\Phi \right)\subset \sigma_{\mathrm{pp}}^{\mathrm{ph}}\left(V_{\varphi ,\Phi }\right)$.

**Proof.**  Fix $\lambda \in \sigma_{\mathrm{pp}}^{\mathrm{ph}}\left(\Phi \right)$, together with a unitary eigenvector $u_{\lambda }\in A_{\lambda }$, which exists by the previous proposition. For $\eta_{\lambda }:=\pi_{\varphi }\left(u_{\lambda }\right)\xi_{\varphi }\in H_{\varphi }$, first, we get $V_{\varphi ,\Phi }\eta_{\lambda }=\lambda \eta_{\lambda }$, and second, $∥\eta_{\lambda }{∥}^{2}=\varphi \left(u_{\lambda }^{*}u_{\lambda }\right)=1$. □

The key-point of our analysis is the following result, which is nothing but the noncommutative version of Lemma 2.1 in [5].

**Lemma** **1.** Consider the uniquely ergodic $C^{*}$-dynamical system (A,Φ,φ), together with a sequence of states ${\left\{\omega_{n}\right\}}_{n\in N}\subset S\left(A\right)$. Then, for each a∈A and $\lambda =e^{-ı\theta }$,

$$\mu_{\pi_{\varphi }\left(a\right)\xi_{\varphi }}{\left(\left\{\theta \right\}\right)}^{1/2}\geq \underset{n}{lim sup}\frac{1}{n}|\sum_{k=0}^{n-1}\omega_{n}\left(\Phi^{k}\left(a\right)\right)\lambda^{-k}|\;.$$

**Proof.**  With $\lambda =e^{-ı\theta }$, consider the $C^{*}$-tensor product C(T)⊗A≡C(T;A) together with the *-homomorphism $\tilde{\Phi }:C\left(T;A\right)\to C\left(T;A\right)$ given by

$$\tilde{\Phi }\left(f\right)\left(s\right):=\Phi \left(f(s+\theta )\right)\;,\;f\in C\left(T;A\right)\;,\;\;\;\theta \in \left[0,2\pi \right)\;.$$

For ${\left\{\omega_{n}\right\}}_{n\in N}\subset S\left(A\right)$, let ${\left\{\tilde{\omega_{n}}\right\}}_{n\in N}\subset S\left(C\left(T;A\right)\right)$ be the sequence of states given by

$$\begin{array}{cc} \tilde{\omega_{n}}\left(f\right):= & \left(\frac{1}{n}\sum_{k=0}^{n-1}\left(\delta_{0}⊗\omega_{n}\right)\circ {\tilde{\Phi }}^{k}\right)\left(f\right)\\ = & \left(\frac{1}{n}\sum_{k=0}^{n-1}\delta_{k\theta }⊗\left(\omega_{n}\circ \Phi^{k}\right)\right)\left(f\right)\\ = & \frac{1}{n}\sum_{k=0}^{n-1}\omega_{n}\left(\Phi^{k}\left(f\left(k\theta \right)\right)\right)\;. \end{array}$$

Notice that for the function $f\left(s\right):=ae^{ıs}\in C\left(T;A\right)$,

$$\tilde{\omega_{n}}\left(f\right)=\frac{1}{n}\sum_{k=0}^{n-1}\omega_{n}\left(\Phi^{k}\left(a\right)\right)\lambda^{-k}\;.$$

Let ${\left\{n_{j}\right\}}_{j\in N}\subset N$ be a subsequence such that

$$\underset{n}{lim sup}\frac{1}{n}|\sum_{k=0}^{n-1}\omega_{n}\left(\Phi^{k}\left(a\right)\right)\lambda^{-k}|=\lim_{j}\frac{1}{n_{j}}|\sum_{k=0}^{n_{j}-1}\omega_{n_{j}}\left(\Phi^{k}\left(a\right)\right)\lambda^{-k}|\;,$$

and consider any *-weak limit point $\tilde{\omega }$ of the sequence ${\left\{\tilde{\omega_{n_{j}}}\right\}}_{j\in N}$, which exists by the Banach Alaoglu theorem, see, e.g., [19], Theorem 4.21. By passing to a subsequence if necessary, we get

$$|\tilde{\omega }\left(f\right)|=\lim_{j}\frac{1}{n_{j}}|\sum_{k=0}^{n_{j}-1}\omega_{n_{j}}\left(\Phi^{k}\left(a\right)\right)\lambda^{-k}|\;.$$

Let ω∈S(A) be the marginal of $\tilde{\omega }$ defined on constant functions $f_{a}\left(s\right):=a$ by

$$\omega \left(a\right):=\tilde{\omega }\left(f_{a}\right)\;,\;a\in A\;.$$

By construction, $\tilde{\omega }$ is invariant under $\tilde{\Phi }$. Therefore, ω is invariant under Φ, as well, which means ω=φ because (A,Φ,φ) is uniquely ergodic.

Let $\left(H_{\tilde{\omega }},\pi_{\tilde{\omega }},V_{\tilde{\omega },\tilde{\Phi }},\xi_{\tilde{\omega }}\right)$ be the covariant GNS representation associated with $\tilde{\omega }$. By computing as in Lemma 2.1 of [5], we then conclude for the spectral measures associated with $V_{\tilde{\omega },\tilde{\Phi }}$ and $V_{\varphi ,\Phi }$,

$$\mu_{\pi_{\tilde{\omega }}\left(f\right)\xi_{\tilde{\omega }}}\left(\left\{0\right\}\right)=\mu_{\pi_{\varphi }\left(a\right)\xi_{\varphi }}\left(\left\{\theta \right\}\right)\;.$$

Therefore, with $P_{\mathrm{const}}\in B\left(H_{\tilde{\omega }}\right)$, the orthogonal projection onto the one-dimensional subspace $C1\;\;I_{C(T;A)}=C⊗1\;\;I_{A}$,

$$\begin{array}{cc} \mu_{\pi_{\varphi }\left(a\right)\xi_{\varphi }}{\left(\left\{\theta \right\}\right)}^{1/2}= & \mu_{\pi_{\tilde{\omega }}\left(f\right)\xi_{\tilde{\omega }}}{\left(\left\{0\right\}\right)}^{1/2}=∥P_{1}\left(f\right)∥\geq ∥P_{\mathrm{const}}\left(f\right)∥\\ = & |\tilde{\omega }\left(f\right)|=\underset{n}{lim sup}\frac{1}{n}|\sum_{k=0}^{n-1}\omega_{n}\left(\Phi^{k}\left(a\right)\right)\lambda^{-k}|\;. \end{array}$$

 □

## 3. The Main Result

The present section is devoted to the following ergodic result we want to prove. Denote with $\chi_{S}$ the characteristic function of the subset S⊂X by

$$\chi_{S}\left(x\right):=\left\{\begin{array}{cc} 1 & \mathrm{if}\;\;x\in S\;,\\ 0 & \mathrm{if}\;\;x\in S^{c}:=X\backslash S\;. \end{array}\right.$$

**Theorem** **1.** Let (A,Φ,φ) be a uniquely ergodic $C^{*}$-dynamical system. Fix $\lambda \in X:=\sigma_{\mathrm{pp}}^{\mathrm{ph}}\left(\Phi \right)⋃\left(T\backslash \sigma_{\mathrm{pp}}^{\mathrm{ph}}\left(V_{\varphi ,\Phi }\right)\right)$. Then, for each a∈A,

$$\lim_{n}\frac{1}{n}\sum_{k=0}^{n-1}\Phi^{k}\left(a\right)\lambda^{-k}=\chi_{\sigma_{\mathrm{pp}}^{\mathrm{ph}}\left(\Phi \right)}\left(\lambda \right)\varphi \left(u_{\lambda }^{*}a\right)u_{\lambda }\;,$$

*uniformly for n→+∞, where $u_{\lambda }\in A_{\lambda }$ is any unitary eigenvalue corresponding to $\lambda \in \sigma_{\mathrm{pp}}^{\mathrm{ph}}\left(\Phi \right)$.*

**Proof.**  First consider the case $\lambda \in \sigma_{\mathrm{pp}}^{\mathrm{ph}}\left(\Phi \right)$, and take a unitary eigenvector $u_{\lambda }\in A_{\lambda }$. Since Φ is multiplicative, we have

$$\varphi \left(u_{\lambda }^{*}a\right)u_{\lambda }=u_{\lambda }\lim_{n}\left(\frac{1}{n}\sum_{k=0}^{n-1}\Phi^{k}\left(u_{\lambda }^{*}a\right)\right)=\lim_{n}\left(\frac{1}{n}\sum_{k=0}^{n-1}\Phi^{k}\left(a\right)\lambda^{-k}\right)\;.$$

Let now $\lambda \notin \sigma_{\mathrm{pp}}^{\mathrm{ph}}\left(V_{\varphi ,\Phi }\right)$, and suppose $\frac{1}{n}\sum_{k=0}^{n-1}\Phi^{k}\left(a\right)\lambda^{-k}↛0$ uniformly. Then, there would exist a sequence of states states ${\left\{\omega_{n}\right\}}_{n\in N}\subset S\left(A\right)$ such that for $\lambda =e^{-ı\theta }$, ${lim sup}_{n}\frac{1}{n}|\sum_{k=0}^{n-1}\omega_{n}\left(\Phi^{k}\left(a\right)\right)\lambda^{-k}|>0$. By Lemma 1,

$$\mu_{\pi_{\varphi }\left(a\right)\xi_{\varphi }}{\left(\left\{\theta \right\}\right)}^{1/2}\geq \underset{n}{lim sup}\frac{1}{n}|\sum_{k=0}^{n-1}\omega_{n}\left(\Phi^{k}\left(a\right)\right)\lambda^{-k}|>0$$

which contradicts $\lambda \notin \sigma_{\mathrm{pp}}^{\mathrm{ph}}\left(V_{\varphi ,\Phi }\right)$. □

We end by constructing simple noncommutative examples for which $\sigma_{\mathrm{pp}}^{\mathrm{ph}}\left(\Phi \right)⊊\sigma_{\mathrm{pp}}^{\mathrm{ph}}\left(V_{\varphi ,\Phi }\right)$ by tensoring a uniquely mixing (see [12]) noncommutative $C^{*}$-dynamical system (B,γ,ω) based on the *-automorphism γ:B→B, with some Anzai skew product (cf. [17]) as those described in Section 3 of [5].

Consider the free group $F_{Z}$ on infinitely many generators ${\left\{g_{j}\right\}}_{j\in Z}$, together with the one-step shift $g_{j}\mapsto g_{j+1}$ acting on the generators. Such a shift induces an action of the group of the integers Z on the reduced group $C^{*}$-algebra $C_{\mathrm{red}}^{*}\left(F_{Z}\right)$ generated by all powers of the corresponding *-automorphism $\gamma \left(\lambda \left(g_{j}\right)\right):=\lambda \left(g_{j+1}\right)$, ${\left\{\lambda (g_{j})\right\}}_{j\in Z}\subset C_{\mathrm{red}}^{*}\left(F_{Z}\right)$ being the unitary generators of the reduced group $C^{*}$-algebra. Here, we have denoted by "λ" the left regular representation of the discrete group $F_{Z}$ on $\ell^{2}\left(F_{Z}\right)$. The left regular representation also realises, up to unitary equivalence, the GNS representation of the reduced group $C^{*}$-algebra associated to the canonical trace.

It was shown in Corollary 3.3 of [12] that the $C^{*}$-dynamical system $\left(C_{\mathrm{red}}^{*}\left(F_{Z}\right),\gamma \right)$ is uniquely mixing, and thus uniquely ergodic with the canonical trace τ as the unique invariant state.

Denote by $\left(H_{\tau },\pi_{\tau },V_{\tau ,\gamma },\xi_{\tau }\right)$ the GNS covariant representation associated with $\left(C_{\mathrm{red}}^{*}\left(F_{Z}\right),\gamma ,\tau \right)$. In particular, we have $\sigma_{\mathrm{pp}}^{\mathrm{ph}}\left(V_{\tau ,\gamma }\right)=\left\{1\right\}$ (Here, we have denoted by “λ” the left regular representation of the discrete group $F_{Z}$ on $\ell^{2}\left(F_{Z}\right)$. The left regular representation also realizes, up to unitary equivalence, the GNS representation of the reduced group $C^{*}$-algebra associated with the canonical trace.).

Let (A,α,φ) be the tensor product $C^{*}$-dynamical system, where

$$A:=C\left(T^{2};C_{\mathrm{red}}^{*}\left(F_{Z}\right)\right)∼C\left(T^{2}\right)⊗C_{\mathrm{red}}^{*}\left(F_{Z}\right)\;,$$

$$\alpha \left(f\right)\left(s\right):=\gamma \left(f(Ts)\right)\;,\;s=\left(s_{1},s_{2}\right)\in T^{2}\;,\;\;f\in C\left(T^{2};C_{\mathrm{red}}^{*}\left(F_{Z}\right)\right)\;,$$

and finally

$$\varphi \left(f\right):=\left(\int \;⊗\tau \right)\left(f\right)=\int_{T^{2}}\tau \left(f\left(s\right)\right)d{\;}^{2}s/4\pi^{2}\;,\;f\in C\left(T^{2};C_{\mathrm{red}}^{*}\left(F_{Z}\right)\right)\;.$$

Here, $s=\left(s_{1},s_{2}\right)\in {\left[0,2\pi \right)}^{2}∼T^{2}$, and $T\left(s_{1},s_{2}\right)=\left(R_{\theta }s_{1},h\left(s_{1}\right)+s_{2}\right)$ is the Anzai skew product corresponding to the rotation $R_{\theta }$ of the angle θ∈[0,2π) such that θ/2π is irrational, and to the continuous function h:T→T.

**Proposition** **3.** If the Anzai skew product T is uniquely ergodic, then the above $C^{*}$-dynamical system (A,α,φ) is uniquely ergodic, as well.

*In addition, there exist Anzai skew products T such that $\sigma_{\mathrm{pp}}\left(\alpha \right)⊊\sigma_{\mathrm{pp}}\left(V_{\varphi ,\alpha }\right)$, and the limit*

$$\lim_{n}\frac{1}{n}\sum_{k=0}^{n-1}\alpha^{k}\left(a\right)\lambda^{-k}$$

*fails to exist in the weak topology, for some a∈A and $\lambda \in \sigma_{\mathrm{pp}}\left(V_{\varphi ,\alpha }\right)\backslash \sigma_{\mathrm{pp}}\left(\alpha \right)$.*

**Proof.**  Since $\left(C_{\mathrm{red}}^{*}\left(F_{Z}\right),\gamma ,\tau \right)$ is uniquely mixing and the Anzai skew product $\left(T^{2},T,d{\;}^{2}s/4\pi^{2}\right)$ is supposed to be uniquely ergodic, by Theorem 3.7 of [20], we argue that (A,α,φ) is uniquely ergodic.

Notice that $\sigma_{\mathrm{pp}}\left(\gamma \right)=\left\{1\right\}=\sigma_{\mathrm{pp}}\left(V_{\tau ,\gamma }\right)$. Therefore, by Lemma 4.17 of [21], each eigenvector $\eta_{\lambda }\in H_{\varphi }=L^{2}\left(T^{2},d{\;}^{2}s/4\pi^{2};H_{\tau }\right)$ corresponding to the eigenvalue $\lambda \in \sigma_{\mathrm{pp}}\left(V_{\varphi ,\alpha }\right)$ is of the form $u_{\lambda }\left(s\right)\xi_{\tau }$ for some unitary function $u_{\lambda }\in L^{2}\left(T^{2},d{\;}^{2}s/4\pi^{2}\right)$ (i.e., a measurable eigenvector in the language of [5]), which is an eigenvector of the Anzai skew-product (i.e., $u_{\lambda }\left(Ts\right)=\lambda u_{\lambda }\left(s\right)$) corresponding to the same value of λ. If moreover, $u_{\lambda }\in C\left(T^{2}\right)$ (i.e., a continuous eigenvector in the language of [5]), then $\lambda \in \sigma_{\mathrm{pp}}\left(\beta_{T}\right)$, $\beta_{T}$ being the dual action of *T* on functions: $\beta_{T}\left(f\right)\left(s\right)=f\left(Ts\right)$. Summarizing, we have

$$\sigma_{\mathrm{pp}}\left(\beta_{T}\right)=\sigma_{\mathrm{pp}}\left(\alpha \right)\subset \sigma_{\mathrm{pp}}\left(V_{\varphi ,\alpha }\right)=\sigma_{\mathrm{pp}}\left(V_{\int ,\beta_{T}}\right)\;.$$

In order to check the latter assertion, it is enough to consider an Anzai skew product and a continuous function $f\in C(T^{2})$ as in Proposition 3.1 of [5], such that

$$\lim_{n}\frac{1}{n}\sum_{k=0}^{n-1}f\left(T^{k}t\right)\lambda^{-k}$$

fails to exist for the point $t\in T^{2}$ and λ∈T. Therefore, for the element $F\left(s\right):=f\left(s\right)1\;\;I_{C_{\mathrm{red}}^{*}\left(F_{Z}\right)}\in A$ and state $\omega :=\delta_{t}⊗\tau \in S\left(A\right)$, we get

$$\lim_{n}\frac{1}{n}\sum_{k=0}^{n-1}\omega \left(\alpha \left(F\right)\right)\lambda^{-k}=\lim_{n}\frac{1}{n}\sum_{k=0}^{n-1}f\left(T^{k}t\right)\lambda^{-k}$$

which fails to exist. □

## 4. Conclusions

The possibility to address various generalizations of ergodic results to the quantum case is usually an improbate task. Concerning the present argument, it was still possible to extend the classical result *mutatis-mutandis* to the quantum situation. However, the following (stimulating in the opinion of the author) problems remain open:

- investigate under which conditions the average (1) still converge, even for $\lambda \in \sigma_{\mathrm{pp}}^{\mathrm{ph}}\left(V_{\varphi ,\Phi }\right)\backslash \sigma_{\mathrm{pp}}^{\mathrm{ph}}\left(\Phi \right)$;
- extend our main result (i.e., Theorem 1) to general positive maps Φ:A→A acting on the $C^{*}$-algebra A;
- provide more complex (i.e., which do not merely come from a tensor product construction) examples for which the average (1) does not converge for some $\lambda \in \sigma_{\mathrm{pp}}^{\mathrm{ph}}\left(V_{\varphi ,\Phi }\right)\backslash \sigma_{\mathrm{pp}}^{\mathrm{ph}}\left(\Phi \right)$.
